# *BMP-2 *gene-fibronectin-apatite composite layer enhances bone formation

**DOI:** 10.1186/1423-0127-18-62

**Published:** 2011-08-23

**Authors:** Wei Zhang, Hideo Tsurushima, Ayako Oyane, Yushin Yazaki, Yu Sogo, Atsuo Ito, Akira Matsumura

**Affiliations:** 1 Nanosystem Research Institute (NRI), National Institute of Advanced Industrial Science and Technology (AIST), Higashi 1-1-1, Tsukuba, Ibaraki 305-8565, Japan; 2 Department of Neurosurgery, Graduate School of Comprehensive Human Science, University of Tsukuba, Tennoudai 1-1-1, Tsukuba, Ibaraki 305-8575, Japan; 3 Institute of Human Science and Biomedical Engineering, National Institute of Advanced Industrial Science and Technology (AIST), Higashi 1-1-1, Tsukuba, Ibaraki 305-8565, Japan; 4 Technical Institute of Physics and Chemistry, Chinese Academy of Sciences, Beijing 100190, China

**Keywords:** bone engineering, *BMP-2 *gene-fibronectin-apatite composite layer, *BMP-2 *gene therapy, non-viral gene transfer.

## Abstract

**Background:**

Safe and efficient gene transfer systems are needed for tissue engineering. We have developed an apatite composite layer including the bone morphogenetic protein-2 (*BMP-2*) gene and fibronectin (FB), and we evaluated its ability to induce bone formation.

**Methods:**

An apatite composite layer was evaluated to determine the efficiency of gene transfer to cells cultured on it. Cells were cultured on a composite layer including the *BMP-2 *gene and FB, and *BMP-2 *gene expression, *BMP-2 *protein concentrations, alkaline phosphatase (ALP) activity, and osteocalcin (OC) concentrations were measured. A bone defect on the cranium of rats was treated with hydroxyapatite (HAP)-coated ceramic buttons with the apatite composite layer including the *BMP-2 *gene and FB (HAP-BMP-FB). The tissue concentration of BMP-2, bone formation, and the expression levels of the *BMP-2, ALP*, and *OC *genes were all quantified.

**Results:**

The apatite composite layer provided more efficient gene transfer for the cultured cells than an apatite composite layer without FB. The BMP-2 concentration was approximately 100~600 pg/mL in the cell-culture medium. Culturing the cells on the apatite composite layer for 27 days increased ALP activity and OC concentrations. In animal experiments, the tissue concentrations of BMP-2 were over 100 pg/mg in the HAP-BMP-FB group and approximately 50 pg/mg in the control groups. Eight weeks later, bone formation was more enhanced in the HAP-BMP-FB group than in the control groups. In the tissues surrounding the HAP button, the gene expression levels of ALP and OC increased.

**Conclusion:**

The *BMP-2 *gene-FB-apatite composite layer might be useful for bone engineering.

## Background

Some gene therapy systems have been reported for bone and cartilage tissue engineering in animal models [1-9]. Bone morphogenetic protein (BMP) genes have often been applied for bone repair, and their usefulness has been reported in various animal experiments [1-5,8]. BMP-2 is a potent osteoinductive factor shown to induce the osteogenic differentiation of mesenchymal cells [10], and treatment systems using recombinant BMP-2 protein show promise for the future. However, these systems using recombinant proteins have several problems, including high doses that range from micrograms up to milligrams (which increases cost) and the short half-life of proteins [11].

A safe and efficient gene transfer system is in high demand in the field of tissue engineering. Gene-apatite particles have long been used as a gene-transferring agent [12-14]. A particulate gene-apatite composite offers increased safety over viral and lipid-based systems, because apatite is the main component of human hard tissues and has both low toxicity and good biocompatibility [15,16]. However, particulate gene-apatite composites have the disadvantage of inefficient gene transfer. To improve its efficiency of gene transfer, a surface-mediated gene transfer system derived from an apatite composite layer was recently developed [17]. We further improved the efficiency of gene transfer by immobilizing cell adhesion molecules [laminin or fibronectin (FB)] in the apatite composite layer [18-20].

Hydroxyapatite has already been applied to various clinically approved bone substitutes to repair bone defects. Hydroxyapatite causes minimal foreign-body reactions and acts as an osteoconductive material by binding to bone [21,22]. Therefore, hydroxyapatite is a good material for use in operations, including cranioplasties, lamioplasties, and cervical anterior fusion. However, it has been reported that a significant amount of time is needed for hydroxyapatite to bind to host bones and achieve osteofusion. It would be ideal for substrates to bind to bone quickly.

We prepared an ethylene-vinyl alcohol copolymer (EVOH) substrate coated by an apatite composite layer that includes both *BMP-2 *gene and FB (EVOH-BMP-FB) for in vitro experiments, and we prepared hydroxyapatite ceramic buttons (HAPs) with the apatite composite layer including the BMP-2 gene and FB (HAP-BMP-FB) for *in-vivo *experiments. The aim was to evaluate the efficiency of gene transfer mediated by this apatite composite layer and the feasibility of using this gene transfer system in bone engineering.

## Materials and methods

### Cell culture

Mouse preosteoblast MC3T3-E1 cells, mouse embryonic mesenchymal cells, C3H10T1/2 cells and human cervical cancer HeLa cells were purchased from RIKEN Bioresource Center (Tsukuba, Japan). MC3T3-E1 and HeLa cells were cultured in minimum essential medium alpha (MEMα; Gibco-BRL, Grand Island, NY, USA) medium including 10% fetal bovine serum (FBS; Thermo Trace, Australia), and C3H10T1/2 cells were cultured in basal medium eagle (BME; Gibco-BRL) including 10% FBS.

### Plasmid construction

The DNA sources used were pGL3 control (Promega Co., Madison, WI, USA) and pCI-neo (Gibco-BRL). pGL3 control includes the cDNA of luciferase. The cDNA of human BMP-2 was inserted into the multiple cloning site of pCI-neo by using EcoRI and NotI sites at the linker ends, and it was named pCI-BMP. The cDNA of BMP-2 was cloned from HeLa cells by reverse transcription PCR. The cDNA was amplified using the following primers: forward primer, 5'-GCGGAATTCGACTGCGGTCTCCTAAAGGTC-3' and reverse primer, 5'- GCGGCGGCCGCTTGCTGTACTAGCGACACCCAC-3'.

### Preparation of substrates

**Figure 1:**
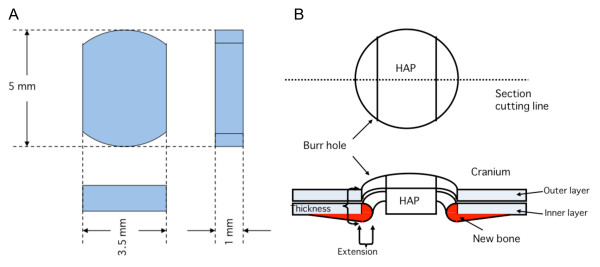
**Three-dimensional views of a hydroxyapatite ceramic button (HAP) and the implantation of HAP samples into bone defects (burr holes)**. A; HAPs were made for cranial repair (cranioplasty) in rats. Both sides of the HAP were cut in order for bone formation to extend into the space around the bone defect. B; The panel demonstrates how bone formation was measured. Bone formation was quantified by measuring the length of new bone extension into the inside of the bone defect and the thickness of the edges of the bone defect.

In *in-vitro *experiments, EVOH with a thickness of 1 mm was obtained by hot-pressing ethylene-vinyl alcohol copolymer pellets (quoted ethylene content of 32 mol%; Kuraray Co., Ltd, Tokyo, Japan). The EVOH was cut into 10 × 10 mm^2 ^square substrates using a level-controlled sample cutter (SDL-200, Dumbbell Co., Ltd, Kawagoe, Japan). The EVOH was abraded on one side with SiC paper (average grain size = 7.6 μm, was ultrasonically washed with acetone and ethanol and was then dried under vacuum for 24 h. HAP buttons were custom-made for the *in-vivo *experiments because it was not easy to form EVOH into the appropriate shape for *in-vivo *experiments and because HAP is a very popular biomaterial [23]. Pure, stoichiometric hydroxyapatite powder was supplemented with 3% (wt. %) polyvinyl alcohol and 1% (wt. %) polyethylene glycol, sieved to select only particles under 75 μm in size, then formed into disks at 98 MPa and sintered at 1150°C for one hour. The resulting shape of the HAP buttons is shown in Figure four A, and each button has a surface area of 15.94 mm^2 ^and a mean thickness of 1.00 mm. The HAP buttons were designed for a round cranial bone defect 5 mm in diameter, and their sides were cut bilaterally to permit bone formation into the space that was created by cutting (Figure 1A).

### Deposition of amorphous calcium phosphate on the surface of the substrate

Unlike HAP, EVOH has no nucleation site for apatite on its surface. Therefore, a surface modification process using amorphous calcium phosphate as a nucleating agent for the apatite was applied to the EVOH prior to the coating process [24-26]. EVOH was used in all of the *in-vitro *experiments. The EVOH was subjected to the following amorphous calcium phosphate-modification process, which was originally developed for an apatite coating process [27]. First, each substrate was dipped into 20 ml of aqueous 200 mM CaCl_2 _(Nacalai Tesque, Inc., Kyoto, Japan) for 10 s, then into ultrapure water for 1 s, and then dried. Each substrate was then dipped into 20 ml of aqueous 200 mM K_2_HPO_4_·3H_2_O (Nacalai Tesque, Inc.) for 10 s, then into ultrapure water for 1 s, and then dried. The alternate dipping into calcium and phosphate ion solutions described above was performed three times. As a result of this process, nanoparticles of amorphous calcium phosphate, which is a precursor of apatite, were deposited onto each EVOH substrate [28].

### Coating process

A calcium phosphate (CP) solution was prepared by dissolving NaCl (final concentration = 142 mM), K_2_HPO_4_·3H_2_O (1.50 mM), 1 M HCl solution (40 mM), and CaCl_2 _(3.75 mM) (Nacalai Tesque Inc.) in ultrapure water and then buffering the solution at pH = 7.40 at 25°C with tris(hydroxymethyl)aminomethane (final concentration = 50 mM) and the necessary quantity of 1 M HCl (Nacalai Tesque Inc.) [29-31]. Coating solutions were prepared by supplementing CP solution with 40 μg/mL of plasmid and/or 20 μg/mL FB. The FB source that was used was 1 mg/mL FB from bovine plasma (Sigma-Aldrich). The plasmid that was used was propagated and purified to a concentration of 0.7-1.2 mg/mL. The EVOH was sterilized by exposure to ethylene oxide gas and then aseptically immersed in 3 mL of the coating solution at 25°C for 24 h. The HAP was sterilized at 180°C for 6 h and immersed in 3 mL of the coating solution at 25°C for 24 h. HAP lacking amorphous calcium phosphate deposition was treated in 2 mL of the coating solution at 25°C for 24 h. The coating for these substrates was performed in CP solution alone or CP solution including plasmid and/or FB. The following materials were prepared:

• EVOH-CP and HAP-CP in CP solution alone.

• EVOH-FB in CP solution supplemented with FB.

• EVOH-DNA in CP solution supplemented with pGL3 control.

• EVOH-DNA-FB in CP solution supplemented with pGL3 control and FB.

• EVOH-BMP and HAP-BMP in CP solution supplemented with pCI-BMP.

• EVOH-BMP-FB and HAP-BMP-FB in CP solution supplemented with pCI-BMP and FB.

The coating solution was clear and showed no apparent spontaneous precipitation during the coating process. The substrate that was removed from the coating solution was gently washed with phosphate-buffered saline prior to the *in-vitro *or *in-vivo *experiments. The immobilized doses of calcium, phosphate, DNA, and FB were estimated by analyzing the residual coating solutions [18-20,23].

### Analysis of the surface of the samples

The surface structures of the samples were examined by scanning electron microscopy (SEM; Model XL30, FEI Company, Netherlands). The amounts of fibronectin and plasmid immobilized on the samples' surfaces were estimated by analyzing the coating solutions by UV-vis spectrophotometry (Model V-550, JASCO Corporation, Japan) for any residual FB and plasmid after the coating. A protein assay kit (Bio-Rad Laboratories Inc., USA) was used to measure the FB concentration.

### *In-vitro *experiments

The cells were seeded into a 24-well cell culture plate at a concentration of 2 × 10^4 ^cells/well with 0.5 mL medium. The cells were cultured on EVOH-CP, EVOH-DNA, EVOH-DNA-FB, EVOH-BMP, or EVOH-BMP-FB for 3 days or 7 days. In some samples, the cells were washed three times with phosphate-buffered saline (PBS) and lysed in 200 μL of cell culture lysis reagent (Promega). After vortexing, the supernatant was obtained by centrifuging. To evaluate the gene transfer efficiency, luciferase activity was measured in cells cultured on EVOH-DNA and EVOH-DNA-FB using a luminometer (Gene Light 55, Microtec, Japan) and a luciferase assay kit (Promega). Cells cultured on EVOH-CP, EVOH-BMP and EVOH-BMP-FB were used to detect *BMP-2 *gene expression.

### *In-vitro *bone development

MC3T3-E1 cells were seeded into a 24-well cell culture plate at a concentration of 2 × 10^3 ^cells/well with 0.5 mL medium. The cells were cultured on EVOH, EVOH-FB, EVOH-BMP, or EVOH-BMP-FB for 7 days or 27 days. The medium was replaced every week. In some samples, the cells were washed three times with phosphate-buffered saline (PBS) and lysed with 200 μL of cell culture lysis reagent (Promega). After vortexing, the supernatant was obtained by centrifuging. Some samples were used to detect alkaline phosphatase (ALP) activity and osteocalcin (OC) concentration.

### Animal experiments

During all of the experiments (which were approved by the Animal Care and Use Committee in The National Institute of Advanced Industrial Science and Technology), the animals were housed and handled in accordance with the guidelines of the National Institutes of Health. Seven- to eight-week-old male Wistar rats were purchased (Japan Crea Co., Ltd., Japan). Under anesthesia, a round craniotomy (5 mm in diameter) was drilled into the right parietal bone. The rats were divided into three treatment groups. In the HAP-CP group, the cranioplasty was performed with HAP-CP alone. The HAP-BMP group was treated with HAP-BMP without FB. The HAP-BMP-FB group was treated with HAP-BMP-FB. The rats were sacrificed at 2 and 8 weeks after the procedures, and the skull bones with the defects or bone defect tissues were removed. The bone samples were fixed in 10% formaldehyde in PBS for 4 days, demineralized in 10% ethylene diamine tetraacetic acid solution at 4°C for 3 days, and then embedded in paraffin and cut into 10- μm-thick sections. The samples were cut into the center of the skull defect (or at the nearest possible site) at a right angle across the lengthwise axis of the HAP button (Figure 1B). These sections were stained with hematoxylin and eosin and viewed using an IX71 microscope system equipped with DP-Controller imaging software (Olympus, Japan). In cranial bone healing, it has been reported that bone formation occurs at the periphery of the bone defect [32] and on the dural membrane side [33]. Bone formation was quantified by measuring the length of new bone extension into the inside of the bone defect and the thickness of the edges of the bone defect using the IX71 microscope system (Olympus) (Figure 1B). In some rats, the gene expression levels of *BMP-2*, *ALP *and *OC *and BMP-2 were evaluated in the tissues inside of the bone defects.

### *BMP-2*, *ALP *and *OC *gene expression

The *in-vitro *cell samples were washed three times with PBS. The samples from the *in-vitro *cells or *in-vivo *tissues were homogenized and centrifuged, and the supernatant was used to extract RNA. Total RNA was extracted from some samples with an RNA extraction kit (QIAGEN). One microgram of total RNA was reverse transcribed in a buffer containing 1 μl of oligo-dT primers (2.5 μM), 250 μM deoxynucleotides, 10 U RNasin (Promega) and 100 U Superscript II (Gibco-BRL). This mixture was incubated for 75 min at 42°C and for 5 min at 75°C. The gene expression levels of *BMP-2, ALP, OC *and *GAPDH *were detected using the following primers: forward primer 5'-GCCAGCCGAGCCAACAC-3' and reverse primer 5'-AAATTAAAGAATCTCCGGGTTGT-3' for human *BMP2*; forward primer 5'-GAGCAGGAACAGAAGTTTGC-3' and reverse primer 5'-GTTGCAGGGTCTGGAGAGTA-3' for mouse *ALP *[34]; forward primer 5'-AGCTCAACCCCAATTGTGAC-3' and reverse primer 5'-AGCTGTGCCGTCCATACTTT-3' for mouse *OC *[34]; and forward primer 5'-AACTCCCATTCCTCCACCTT-3' and reverse primer 5'-GAGGGCCTCTCTCTTGCTCT-3' for mouse *GAPDH *[34]. Each primer (12.5 pM) was added to a solution containing 12.5 μl of iQ SYBR green supermix (Bio-Rad Laboratories) along with 0.5 μl of template sample (final volume, 25 μl). The Mini Opticon real-time PCR system (Bio-Rad Laboratories Inc.) was used. The gene expression levels were expressed either as the delta cycle time (Δ C(t)) or the delta-delta cycle time (Δ-Δ C(t)), and values normalized to GAPDH expression were compared with the gene expression in HAP-CP.

### BMP-2 and OC protein concentrations and ALP activity

The cell-culture medium was used to measure the concentration of BMP-2 protein using the human/mouse/rat BMP-2 Quantikine ELISA kit (R&D Technologies Inc. RI, USA). Cells cultured on the substrate were lysed by freezing and thawing for three cycles in 200 μl of PBS including 1% TritonX-100. Then, the cell lysis solution was centrifuged at 12,000 g for 2 min at 4°C. The supernatant was used to measure ALP activity using a LabAssay ALP activity kit (Wako Pure Chemical Industries, Ltd., Japan). Protein was quantified in the cell lysis supernatants using a micro-BCA protein assay kit (Thermo Fisher Scientific Inc., MA, USA). The concentration of OC protein in the culture medium was measured using a rat osteocalcin enzyme immunometric assay kit (Biomedical Technologies Inc., USA). The tissues inside the bone defect were homogenized in 400 μl of PBS including 1% Triton X-100, and then, the cell lysate solutions were centrifuged. The supernatant was used to measure the concentration of BMP-2 protein using a human/mouse/rat BMP-2 Quantikine ELISA kit (R&D Technologies Inc.). Protein was quantified in the supernatant using a micro-BCA protein assay kit (Thermo Fisher Scientific Inc.).

### Statistical analysis

The experimental results are expressed as the mean ± the standard deviation. All data were analyzed using Student's t-test, and probability values less than 0.05 were considered to be statistically significant.

## Results

### Surface evaluation

**Figure 2:**
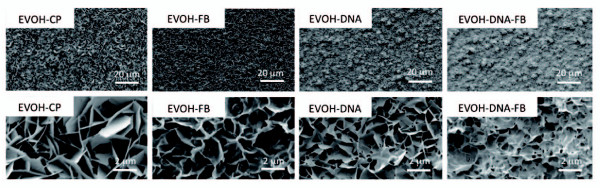
**SEM photos of the EVOH-CP, EVOH-FB, EVOH-DNA and EVOH-DNA-FB substrates**. Uniform layers were observed on the surfaces of all the samples. High magnification images (the lower micrographs) show that all these layers have a microflake-like architecture.

SEM and UV-vis results revealed that composite layers containing apatite had formed on EVOH and HAP treated in CP solution supplemented with plasmid and/or FB. A plasmid/FB/apatite composite layer formed in CP solution supplemented with 40 μg/mL plasmid and 10 μg/mL FB, a plasmid/apatite composite layer formed in CP solution with 40 μg/mL plasmid, an FB/apatite composite layer formed in CP solution with 10 μg/mL FB, and an apatite layer formed in CP solution alone. As shown in the SEM images of EVOH in Figure 1, uniform layers were observed on the surfaces of all the samples. High magnification images (lower micrographs) show that all the layers had microflake-like architecture (Figure 2). The calcium dose, phosphate dose, plasmid content, and FB dose on the sample's surface were measured (Table 1).

**Table 1:** The immobilized doze of calcium, phosphate, DNA and FB

	Ca (μg/cm^2^)	P (μg/cm^2^)	DNA (μg/cm^2^)	Fibronectin (μg/cm^2^)
EVOH-CP	119.7 ± 4.385	69.28 ± 2.501	-	-

EVOH-FB	112.9 ± 8.051	57.57 ± 3.265	-	12.30 ± 0.295

EVOH-DNA	86.57 ± 6.841	45.03 ± 1.399	12.22 ± 0.287	-

EVOH-DNA-FB	108.2 ± 3.021	57.64 ± 3.370	14.00 ± 0.579	17.84 ± 1.332

	Ca (μg/cm^2^)	P (μg/cm^2^)	DNA (μg/cm^2^)	Fibronectin (μg/cm^2^)

HAP-CP	156.4 ± 8.187	67.99 ± 4.043	-	-

HAP-BMP	205.9 ± 11.87	68.21 ± 7.473	16.04 ± 0.905	-

HAP-BMP-FB	158.6 ± 13.63	42.09 ± 2.820	18.62 ± 2.701	19.60 ± 1.327

### *In-vitro *evaluation of gene expression

**Figure 3:**
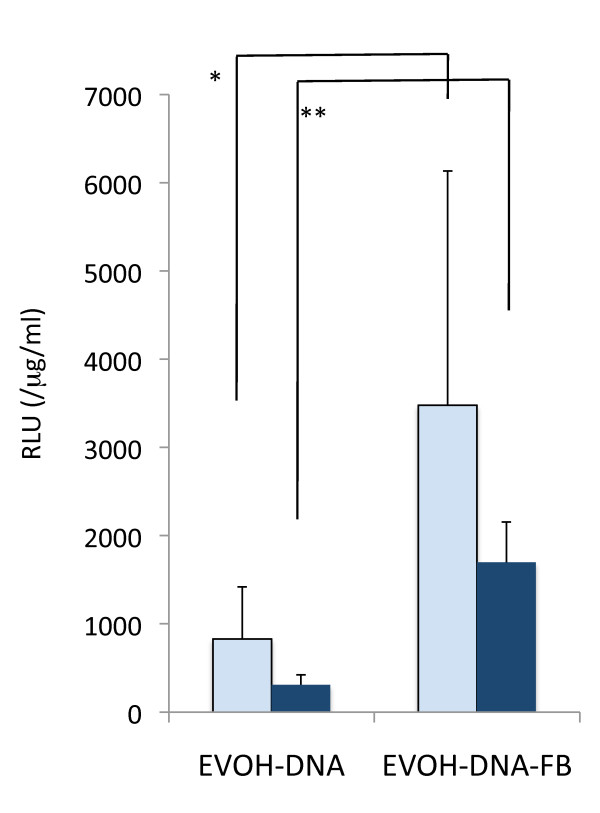
**Relative luciferase assay**. Relative luciferase light units (RLUs, normalized to the protein concentration) of extracts from MC3T3-E1 cells (empty columns) or C3H10T1/2 cells (solid columns) cultured on EVOH-DNA and EVOH-DNA-FB for 3 days. The values presented are the mean ± standard deviation. (n = 3, *p < 0.05, **p < 0.001).

**Figure 4:**
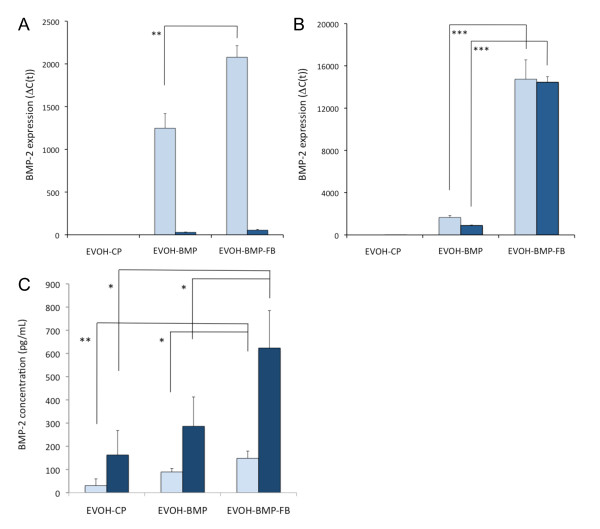
***BMP-2 *gene expression levels and BMP-2 protein concentrations in *in-vitro *experiments**. *BMP2 *gene expression levels in extracts from MC3T3-E1 cells (A) or C3H10T1/2 cells (B) cultured on EVOH-CP, EVOH-BMP, or EVOH-BMP-FB for 3 or 7 days. The empty columns indicate a 3-day culture and the solid columns a 7-day culture. (C) BMP-2 concentrations in the medium from cells cultured on EVOH-CP, EVOH-BMP or EVOH-BMP-FB for 7 days. The empty columns indicate the MC3T3-E1 cells and the solid columns the C3H10T1/2 cells. The values presented are the mean ± standard deviation. (n = 3, *p < 0.05, **p < 0.01, ***p < 0.001).

MC3T3-E1 and C3H10T1/2 cells were cultured on EVOH-DNA and EVOH-DNA-FB with pGL3 control DNA for 3 days, at which time luciferase assays were performed. In both the MC3T3-E1 and C3H10T1/2 cells, the relative luciferase units (RLUs) were a few times higher after growth on EVOH-DNA-FB than on EVOH-DNA (Figure 3). Next, the pGL3 control was switched to pCI-BMP, and the cells were cultured on each substrate for 3 days or 7 days. After 3 days, *BMP-2 *expression was a few fold higher in both cell lines after growth on EVOH-BMP-FB compared with EVOH-BMP (Figure 4A, B). After 7 days, numerous MC3T3-E1 cells had detached from both EVOH-BMP-FB and EVOH-BMP due to cell confluence, and *BMP-2 *expression could not be evaluated (Figure 4A). Some C3H10T1/2 cells had detached and *BMP-2 *expression remained at the same level as that of the 3-day samples (Figure 4B). BMP-2 concentrations were measured in the 3 day-culture medium from both EVOH-BMP and EVOH-BMP-FB. The BMP-2 concentration increased to over 600 pg/mL in the C3H10T1/2 cell-cultured medium (Figure 4C). These findings suggested that the presence of FB enhanced gene transfer in both the EVOH-BMP-FB and EVOH-DNA-FB substrates, and gene expression maybe sustained for one week.

### *In-vitro *bone development

**Figure 5:**
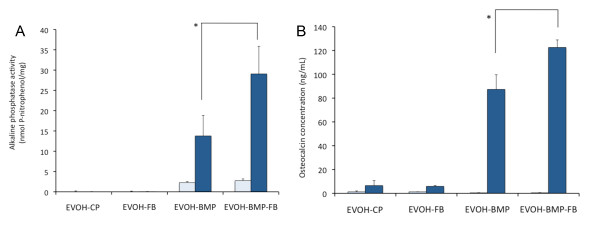
**Development of MC3T3-E1 cells in *in-vitro *experiments**. A; Alkaline phosphatase activity of cells cultured on EVOH-CP, EVOH-FB, EVOH-BMP or EVOH-BMP-FB for 9 or 27 days. B; Osteocalcin concentration of cells cultured on EVOH-CP, EVOH-FB, EVOH-BMP or EVOH-BMP-FB for 9 or 27 days. The empty columns indicate a 9-day culture and the solid columns a 27-day culture. The values presented are the mean ± standard deviation. (n = 3, *p < 0.01).

Bone induction in the MC3T3-E1 cells cultured on each substrate was evaluated by measuring ALP activity and OC protein levels. The MC3T3-E1 cells were cultured for 9 and 27 days and each assay was performed. In the cells grown on EVOH-BMP-FB, ALP activity increased with culturing time and was significantly higher than that in cells grown on EVOH-BMP at Day 27 (Figure 5A). OC levels were significantly higher when the cells were grown on EVOH-BMP-FB than on EVOH-BMP (Figure 5B). These findings indicate that BMP-2 expressed by gene transfer from EVOH-BMP or EVOH-BMP-FB maintains its biological activity and induces bone development in MC3T3-E1 cells.

### *In-vivo *gene transfer

**Figure 6:**
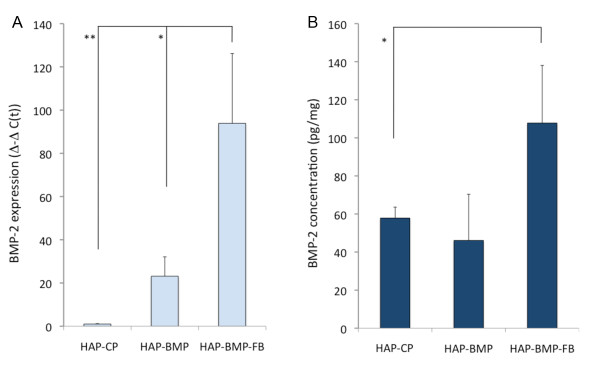
***BMP-2 *gene expression and BMP-2 protein concentrations in animal experiments**. *BMP-2 *gene expression (A) and BMP-2 protein concentrations (B) were evaluated in bone defect tissue treated with HAP-CP, HAP-BMP or HAP-BMP-FB two weeks after the procedure. The values presented are the mean ± standard deviation (n = 3, *p < 0.05, **p < 0.01).

Bone defect rat models treated with HAP-CP, HAP-BMP, or HAP-BMP-FB were sacrificed 2 weeks after the procedure (n = 3 for each group). The tissues in the bone deficit were taken, *BMP-2 *gene expression was evaluated with real-time PCR using primers specific to human *BMP-2 *and BMP-2 concentrations in the tissues were assessed using the human/mouse/rat BMP-2 Quantikine ELISA kit (R&D Technologies Inc.). *BMP-2 *gene expression was higher in the tissues treated with HAP-BMP-FB than in those treated with HAP-BMP or HAP-CP (Figure 6A). The BMP-2 concentration was approximately 108 pg/mg in HAP-BMP-FB, which was higher than that in HAP-BMP or HAP-CP (Figure 6B). These results suggest that the *in-vivo *gene transfer ability of HAP-BMP-FB is higher than that of HAP-BMP.

### *In-vivo *bone development

**Figure 7:**
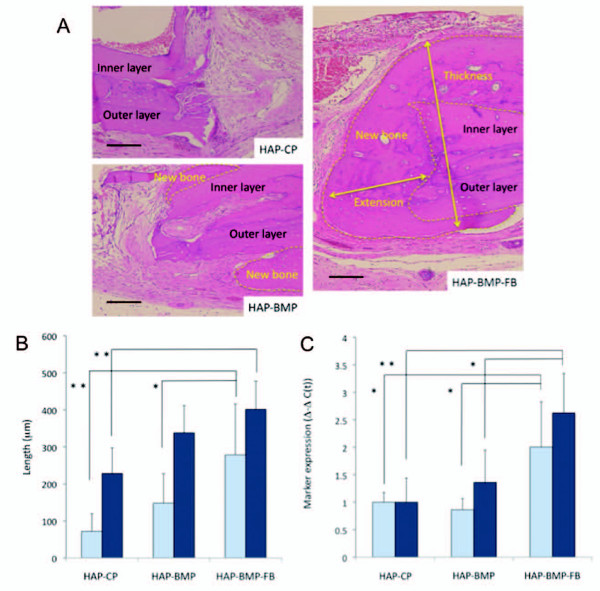
**Evaluation of the animal experiments**. A; Histological sections of the bone defects were stained with hematoxylin and eosin after demineralization. The bone defects were treated with HAP-CP, HAP-BMP or HAP-BMP-FB 8 weeks ago. The yellow dotted lines show the area of bone formation (indicated by new bone). Bone formation was observed between the cranium and the dural membrane, resulting in increased cranial thickness. Bone formation was also observed in the bone defect space as the extension of new bone. Bars indicate 100 μm. B; Bone formation was quantified in each group. The extension of new bone into the space left by the bone defect (open columns). The increased thickness of the cranium due to the bone formation (solid columns). The values presented are the mean ± standard deviation. (n = 5, *p < 0.05, **p < 0.01) C; *ALP *and *OC *gene expression in the bone defect tissue 8 weeks after the procedure. Open columns indicate *ALP *gene expression and solid columns indicate *OC *expression. The values presented are the mean ± standard deviation (n = 5, *p < 0.05, **p < 0.01).

The rat models with a bone deficit treated with HAP-CP, HAP-BMP, or HAP-BMP-FB were sacrificed 8 weeks after the procedure (n = 5 for each group). Bone formation was quantified by measuring the length of new bone extension into the inside of the bone defect and the thickness of the edges of the bone defect [23]. Small pieces of tissues in the bone defect were taken, and the expression levels of the *ALP *and *OC *genes were evaluated. Figure 7A shows histological sections of bone formation at the edge of the cranium in the bone defect. In the HAP-BMP-FB group, bone formation was enhanced significantly more than in the HAP-BMP and HAP-CP groups (Figure 7B). The expression levels of the *ALP *and *OC *genes increased more in the HAP-BMP-FB group than in the HAP-BMP or HAP-CP groups (Figure 7C). These findings suggest that HAP-BMP-FB enhances bone formation more than HAP-BMP or HAP-CP.

## Discussion

Non-viral gene transfer systems are easier to use and safer than viral gene transfer systems, but it is difficult to obtain a high gene transfer ratio [35,36]. Low gene transfer ratios have limited the application of non-viral gene transfer systems. Cytokines require an effective concentration to exert their biological effects, and cytokine production is a component of certain gene therapies. Therefore, we have been trying to improve the gene transfer ratio of our non-viral gene transfer systems [18-20]. Some non-viral gene transfer systems exhibit a degree of cytotoxicity because certain of their components (such as phospholipids) are administered *in-vivo *in high amounts. The cytotoxicity of the components of non-viral gene transfer systems must be taken into account. The elements used in our gene transfer system are DNA, calcium phosphate, and adhesion protein, which are thought safe. In this study, we evaluated whether our system provides gene transfer ratios high enough to have biological effects and thus to have potential for *in-vivo *applications.

Nie et al. reported a BMP-2 gene therapy system that uses DNA/chitosan nanoparticles [37]. In this study, the successful case in which bone formation was enhanced showed serum BMP-2 levels of approximately 3.5 ng/mL instead of approximately 1 ng/mL in the control case. The biologically effective concentration of BMP-2 protein was reported to be over 4.3 ng/mL, and it can act in a dose-dependent manner [38]. Our gene transfer system achieved 108 pg/mg of BMP-2 protein in tissue, a level roughly twice that observed with HAP-CP. Even if successful, in non-viral gene therapy the therapeutic protein concentration might only increase to several times that of the control. Our *BMP-2 *gene-FB-apatite composite layer might stimulate osteoblasts *in-vivo*. Indeed, our experiments indicated that HAP-BMP-FB enhanced bone formation in. In some studies using slow-releasing BMP-2 protein systems, micrograms of proteins were immobilized in a slow-releasing material, which might be too much considering its biologically effective concentration [39,40]. Indeed, ectopic bone formation and bony overgrowths were induced in one such clinical trial, which might have been due to the overdose. Our system induces BMP-2 protein at low concentrations and thus might not have the toxicity and resulting side effects. Systems with high antigenicity, such as adenovirus vector systems, can induce inflammation, which influences tissue regeneration. We thought that the low toxicity of the applied system was an important factor for tissue engineering. Our gene transfer system consists of phosphate, calcium, plasmid DNA and FB, which all have low toxicity. Histological examination revealed no inflammation and no necrosis, indicating that our gene transfer system has good tissue compatibility. Thus, this system has promise for *in-vivo *applications and merits further evaluation.

We have researched the incorporation of functional molecules (such as genes and proteins) into apatite composite layers and the addition of such molecules to the surface of substrates coated with an apatite layer. The ability of incorporated FB to affect gene transfer efficiency is described in our previous report [18-20]. Briefly, cell adhesion molecules (such as FB or laminin) incorporated into a gene-apatite composite layer enhance cell adhesion and cell spreading on the surface of the layer, thereby enlarging the contact area between the cell and the layer. Because of the tight binding between the cell adhesion molecule ligands and the receptors on the cell surface, a stagnant microenvironment is produced at the enlarged contact area between the cell and the layer. The resulting microenvironment is gradually enriched with DNA molecules that are released from the layer. As a result, highly efficient gene transfer is accomplished at the cell adhesion molecule-gene apatite composite layer. In this study, HAP-BMP-FB tightly bound to cells, perhaps mostly fibroblasts, in the surrounding tissues and transferred the BMP-2 gene. *BMP-2 *gene expression was detected for one week in *in-vitro *experiments and for 2 weeks in *in-vivo *experiments, which might indicate that our gene transfer system slowly releases the DNA. However, our other reports have shown that the expression of transferred genes peaks from 3 days to 7 days in *in-vitro *experiments [18-20]. It was unclear when the gene expression peaked in the *in-vivo *experiments. As bone formation was observed in the *in-vivo *experiments despite only a two-fold increase in BMP-2 levels in the HAP-BMP-FB group over the HAP-BMP or HAP-CP group, the peak BMP-2 concentration might occur at an early stage and its value might be higher. Additional pharmaco-dynamic evaluations should be performed in the future. Considering that cytokines would have to be administered for an extended period to develop tissue progenitor cells, a slow releasing gene would be convenient in tissue engineering. Induced paracrine secretion of BMP-2 protein in the bone defect could stimulate the surrounding osteoblasts. Our treatment system would be useful in bone engineering. However, longer-term experiments using animals should be planned to further evaluate the speed and quality of bone formation, because twenty-four weeks might be necessary for cranial defects to completely heal in this rat model [41].

We hope that the apatite composite layer including plasmid and FB might be applied for cranioplasty. In the future, the use of our treatment system in biomaterials could facilitate bone fusion at early stages after cervical operations.

## Conclusion

The *BMP-2 *gene-FB-apatite composite layer was able to enhance bone formation and may be useful for bone engineering. Our gene transfer system might be a useful tool for tissue engineering applications, because it has the potential to control cell differentiation and is both safe and highly efficient.

## Competing interests

The authors declare that they have no competing interests.

## Authors' contributions

WZ, HT and AM conceived of the study, participated in its design and coordination, and helped to draft the manuscript. AO and YY studied the gene-fibronectin-apatite composite layer. YS and AI prepared the hydroxyapatite buttons that were used in the animal experiments. All authors read and approved the final manuscript.
